# Boston Hospital Tent Service

**Published:** 1896-01-18

**Authors:** 


### BOSTON CITY HOSPITAL TENT SERVICE.

Tent hospitals, by reason of their superior airiness and free ventilation, are admittedly the most healthy form of temporary accommodation for the sick in warm dry weather. It is therefore an altogether wise step on the part of the Boston City Hospital to have established a "tent service" for summer use within the grounds of their institution, thus relieving some of the pressure on the already over-full wards, and also allowing necessary repairs and cleaning to be undertaken without diminishing the bed capacity of the hospital, on which such unceasing demands are made.

Thus during the summer ward after ward can be vacated, the patients being removed to the tents in the garden (a change which they probably enjoy), while a thorough overhauling of walls and floors, &c., is carried out within the building. For sudden emergencies, too, such an arrangement has many advantages. The snowy tents on the well-kept turf look cosy and comfortable enough to suggest the thought that the patients might even look forward with pleasure to a sojourn therein.

